# Solamargine acts as an antiviral by interacting to MZF1 and targeting the core promoter of the hepatitis B virus gene

**DOI:** 10.18632/aging.206047

**Published:** 2024-08-10

**Authors:** Wenwen Chen, Xinrui Zhao, Yingli Huang, Kai Lu, Yuan Li, Xiaofang Li, Hui Ding, Xiuling Li, Suofeng Sun

**Affiliations:** 1 Department of Gastroenterology, Zhengzhou University People’s Hospital, Henan Provincial People’s Hospital, Zhengzhou, Henan 450003, China; 2 Master of Chinese medicine (studies and applications of internal Chinese medicines), Hong Kong Baptist University, Kowloon Tong, Hong Kong; 3 Xinxiang Medical University, Clinical Medicine College, Xinxiang, Henan 453000, China; 4 The Third Affiliated Hospital Affiliated of Henan University of Traditional Chinese Medicine, Zhengzhou, Henan 450003, China

**Keywords:** hepatitis B virus, Solamargine, core promoter, MZF1, anti-HBV activity

## Abstract

Background: Hepatitis B virus (HBV) infection is still a serious threat to global health and can lead to a variety of liver diseases, including acute and chronic hepatitis, liver cirrhosis, liver failure, hepatocellular carcinoma (HCC), and so on. At present, there are mainly two kinds of drugs for the treatment of hepatitis B at home and abroad: interferon (IFN) and nucleoside/nucleotide analogs (NAs). In recent years, natural compounds have been considered an important source for the development of new anti-HBV drugs due to their complex structure, diverse components, high efficiency, and low toxicity. Many studies have demonstrated that Solamargine has significant anticancer activity, but the antiviral effect is rarely studied. This study aimed to verify the anti-HBV effect of Solamargine and to explore the specific mechanism.

Method: The relative expression of HBV pregenomic RNA (pgRNA) was detected by reverse transcription real-time fluorescence quantitative PCR (RT-qPCR). Northern blot and western blot were used to detect the relative expression of HBV pgRNA and target protein. PCR was used in the construction of HBV pg-promoter, ENII/BCP, and a series of gene deletion mutant fluorescent reporter vectors. The fluorescence relative expression of each mutant was detected by Renilla luciferase assay.

Results: By binding to MZF1 (Myeloid zinc finger protein 1, MZF1), Solamargine inhibits HBV core promoter activity, reduces pregenomic RNA level, and inhibits HBV, achieving antiviral effects.

## INTRODUCTION

Viral hepatitis type B (HBV) is an infectious disease caused by the Hepatitis B virus (HBV), which mainly causes both acute hepatitis and chronic infection. Furthermore, chronic infection may lead to liver fibrosis, cirrhosis, liver failure, primary Hepatocellular carcinoma (HCC), and so on. The World Health Organization estimated that about 296 million people worldwide were chronically infected with the hepatitis B virus, with about 1.5 million new infections each year, of which about 820,000 died from hepatitis B-associated cirrhosis and hepatocellular carcinoma [1]. According to the latest statistics, there are about 86 million hepatitis B patients in China, about 900,000 new cases of hepatitis B in 2020, and about 350,000 people die every year due to complications related to hepatitis B [2]. In short, HBV-associated liver disease is still a heavy public health burden all around the world.

HBV is a hepatotropic DNA virus, and the genome structure is unique and precise, 3.2 kb long partially double-stranded circular DNA (rcDNA) [3], consisting of a complete coding chain negative chain (long chain) and incomplete non-coding chain positive chain (short chain). HBV transcription is controlled by four promoters and two enhancer elements. The four promoters include the core, preS1 (Sp1), preS2 (Sp2), and X promoter [4]. Core promoter plays a key role in HBV gene expression. It contains Basal core promoter (BCP) and Upper regulatory region (URR). BCP controls the Pre-core RNA (pcRNA) and the Pregenomic RNA (pgRNA) (3.5 kb) [5]. Pro-core RNA encodes pronuclear proteins, called HBeAg. PgRNA not only can be used as templates for viral reverse transcription, translated into core proteins and polymerases but also can be packaged into nucleated capsids as pregenomic RNA intermediates. Enhancer I (ENI) and Enhancer II (ENII), which are upstream of core promoter and regulate the activity of promoters, are dependent on host transcription factor (TF) [5]. ENII is located in nt 1685-1773, immediately upstream of the BCP. ENII regulates viral promoter activity in a BCP-dependent manner and interacts with liver-enriched transcription factors (including nuclear receptor superfamilies) to promote HBV transcription and replication [6].

At present, there are two main types of antiviral therapy for chronic hepatitis B: Interferon (IFN) and Nucleoside/nucleotide analogs (NAs). Interferon therapy is divided into ordinary interferon and Peg-interferon Alfa (PEG-IFN-α), which mainly inhibits viral replication by regulating the immune function of the body, and has direct antiviral effects [7]. NAs, including Lamivudine, Tibivudine, Adefovir, Entecavir, and Tenofovir, work mainly by targeting DNA polymerase to destroy pgRNA reverse transcription [8]. Although they can improve liver inflammation and reduce HBV DNA levels to a certain extent to delay disease progression and reduce the incidence of HCC [9], they cannot guarantee serum HBsAg clearance nor eliminate viral cccDNA of HBV. Therefore, it is necessary to develop new antiviral therapy for chronic hepatitis B.

Natural compounds have the characteristics of novel and diverse structure, low toxicity, high efficiency, and so on, and play an important role in regulating immunity, anti-inflammatory, anti-tumor, antiviral, and antioxidant [10]. Both in the past and the future, natural compounds are an important source of new drugs, and many existing drugs are natural compounds or their derivatives [8]. At present, more and more researchers pay attention to natural products, and many natural drugs with novel structures and anti-HBV activity have been reported. Solamargine is a steroidal alkaloid isolated from Solanum plants, which can be derived from the steroid Solenodon and consists of the basic structural skeleton Spiros Tane and glycoside side chain [11]. Many studies have demonstrated that Solamargine has significant anticancer activity and explored its mechanism of action, but the antiviral effect of Solamargine is rarely studied [12]. The purpose of this study is to screen natural compounds with anti-HBV activity, further explore their anti-HBV targeting sites and specific mechanisms of action, and develop new ideas for safe and effective anti-HBV drug research and development.

## RESULTS

### Solamargine and other natural compounds decreased the relative expression of HBV pgRNA

**Figure 1:**
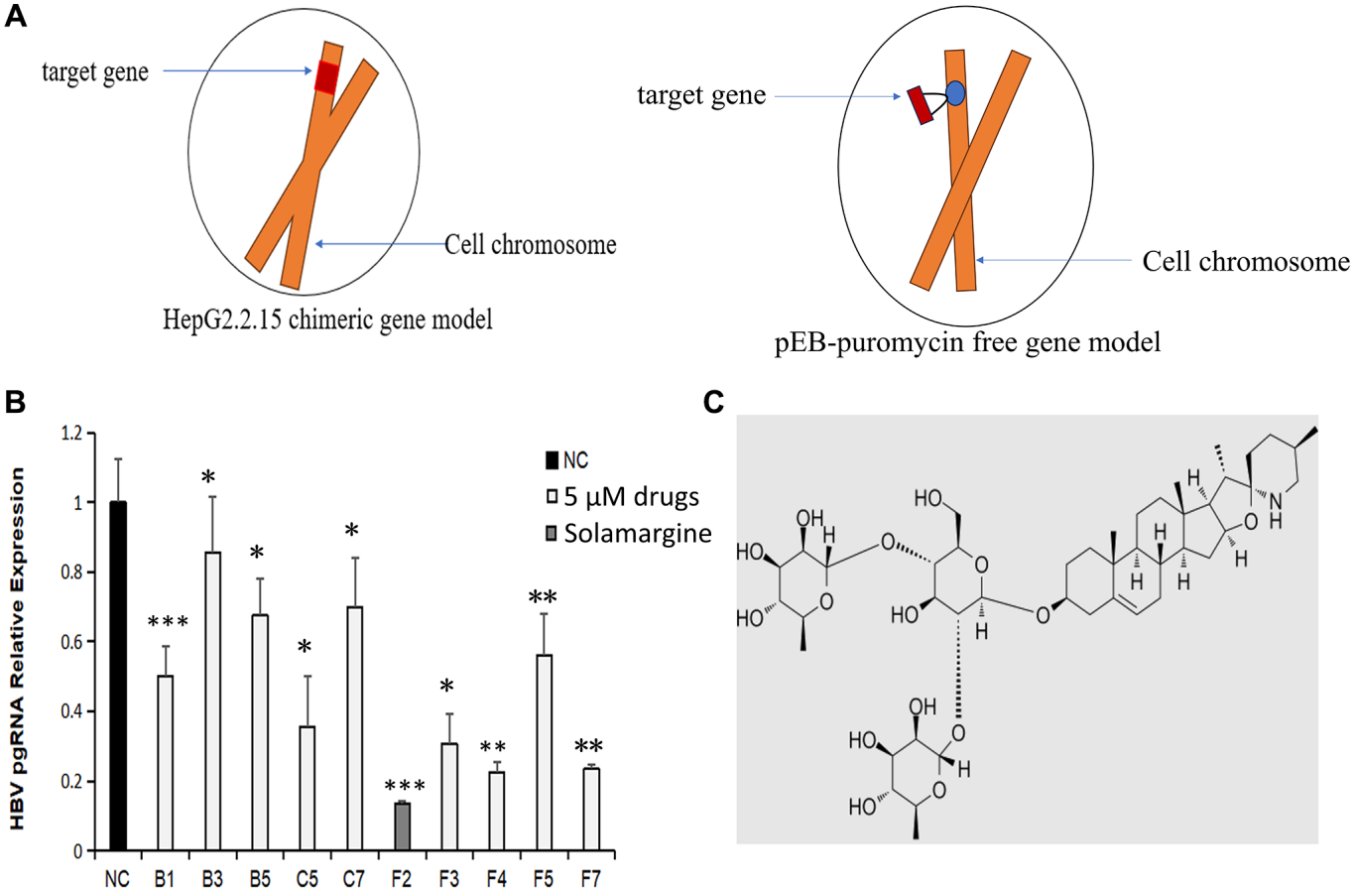
**Effects of Solamargine and other natural compounds on the relative expression of HBV pgRNA.** (**A**) Left: HepG2.2.15 cell model commonly used for *in vitro* studies of HBV; right: pEB-puromycin HBV genome stable expression cell line model constructed in this experiment. (**B**) Huh7 stable cell lines were fused to 90% and then placed on the plates. Afte 1d, natural compounds (5 μM) were added to the experimental groups. After 3d, the cells were collected and total RNA was extracted. The expression level of HBV pgRNA in the cells was detected by RT-qPCR. The quantitative expression level of HBV pgRNA in control group was 1. (**C**) Solamargine molecular structure. The results are means ± SD of 3 independent experiments. ^*^p < 0.05, ^**^p < 0.01, ^***^p < 0.001, Student’s *t*-test.

We purchased a library containing 1200 natural compounds from MCE Company. Through the literature search, we found that the following 10 compounds have varying degrees of antiviral effect, but the inhibition effect on HBV has never been reported (Table 1). To understand the anti-HBV activity of these 10 compounds, we conducted a series of studies. Firstly, the HBV genome stable expression cell line was successfully established based on the pEB-puromycin vector (Figure 1A), The left figure in Figure 1A shows the commonly used HepG2.2.15 chimeric gene model and the right one in Figure 1A shows the free gene model of pEB-puromycin constructed by our research group, which can stably replicate the HBV genome of genotype Ce and is closer to the real model of human infection with HBV, which is more conducive to drug screening experiments. Secondly, the ten natural compounds with anti-HBV activity were screened by RT-qPCR. The primers’ names and sequences that we used are shown in Table 2. The relative content of HBV pgRNA in the control group and the experimental group was detected after 3 days of dosage. The expression level of HBV pgRNA in the experimental group was lower than that in the control group (*P* < 0.05). The Solamargine group (F2) had the lowest value (*P* < 0.001) (Figure 1B, 1C and Supplementary Figure 1). The results showed that all ten natural compounds inhibited the expression of HBV pgRNA, and Solamargine had the most significant inhibitory effect.

**Table 1:** Introduction of 10 compounds.

**No.**	**Product name**	**M.Wt**	**Target**
B1	Hypericin	504.44	Apoptosis; Influenza Virus
B3	Aloin B	418.39	Others
B5	Gypenoside XVII	947.15	Estrogen Receptor/ERR; Endogenous Metabolite
C5	2-(Methylamino)-1H-purin-6(7H)-one	165.15	Bacterial; DNA/RNA Synthesis; Endogenous Metabolite
C7	1-Methyl-L-histidine	169.18	Endogenous Metabolite
F2	Solamargine	868.06	P-glycoprotein; Apoptosis
F3	4-Aminohippuric acid	194.19	Endogenous Metabolite
F4	Palmitelaidic Acid	254.41	AMPK; PPAR; Glucokinase
F5	Lithocholic acid	376.57	Autophagy; Endogenous Metabolite; Apoptosis
F7	Ibotenic acid	158.11	iGluR

**Table 2:** The primers’ names and sequences used in the experiment.

**Name**	**Sequence (5′–3′)**
pgRNA F primer	5′-TCCCTCGCCTCGCAGACG-3′
pgRNA R primer	5′-GTTTCCCACCTTATGAGTC-3′
β-actin F primer	5′-TTCTACAATGAGCTGCGTGTG-3′
β-actin R primer	5′-GGGGTGTTGAAGGTCTCAAA-3′

pgRNA product length (101 bp): TCCCTCGCCTCGCAGACGAAGGTCTCAATCGCCGCGTCGCAGAAGATCTCAATCTCGGGAATCTCAA TGTTAGTATCCCTTGGACTCATAAGGTGGGAAAC.

### Solamargine inhibited the relative expression of HBV DNA

**Figure 2:**
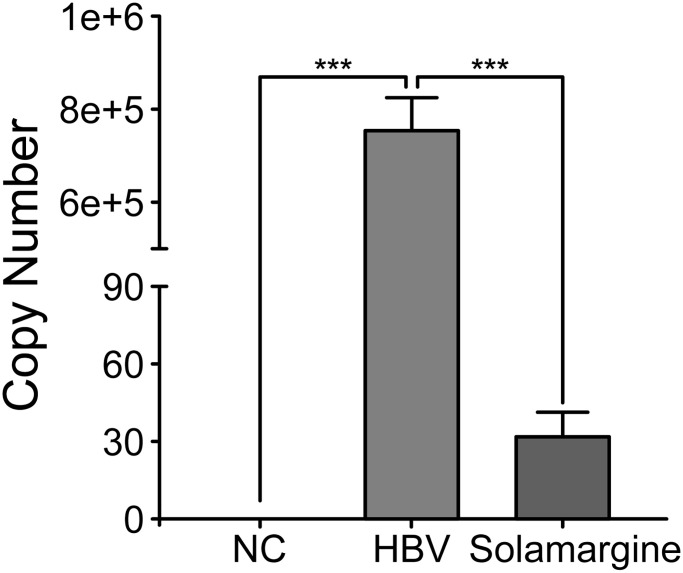
**Effect of Solamargine on the relative expression of HBV DNA in cell supernatants.** HBV plasmid was transfected into Huh7 cells, and 2 d later Solamargine (5 μM) was added into the cells. Samples were collected after 1 d culture and HBV DNA copy number of supernatant was detected by PCR. All assays were performed in triplicate and results are presented as means ± SD. ^***^P < 0.001, Student’s *t*-test.

We initially screened Solamargine, the compound with the most significant anti-HBV activity, so we conducted the following experiments to determine the effect of this compound on HBV. Firstly, the whole HBV genome plasmid was transfected into Huh7 cells. Then, the groups were divided according to the control group (blank group), HBV group (transfected with HBV whole genome plasmid), and Solamargine group (transfected and added 5 μM Solamargine). Then the HBV DNA copy number of supernatant in the three groups was detected by PCR, and the 2^−ΔΔCt^ method was used to calculate the results. (Figure 2). The results showed that Solamargine could effectively inhibit the expression of HBV virus particles in the supernatant of HBV-infected cells.

### Solamargine decreased the relative expression of HBV pgRNA and HBcAg protein

**Figure 3:**
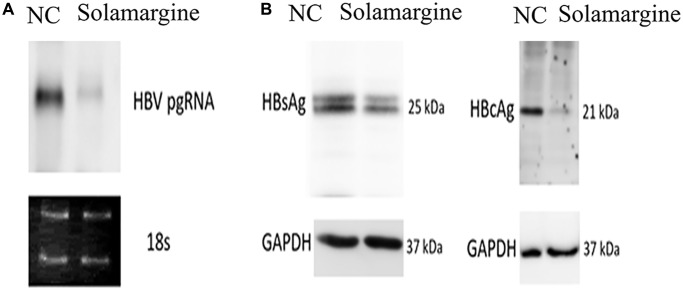
**Effects of Solamargine on the relative expression levels of HBV pgRNA, HBsAg and HBcAg proteins.** HBV plasmid was transfected into Huh7 cells; 2 d later, Solamargine (5 μM) was added into the cells, and the samples were collected after 1 d culture. (**A**) The relative expression of HBV pgRNA was detected by Northern blot. (**B**) The relative expression levels of HBsAg and HBcAg protein were detected by Western blot. The band strength was measured by Image-J software. Assays were performed in triplicate and results are presented as means ± SD.

Northern blot and Western blot were used to further confirm the effects of Solamargine on the relative expression levels of HBV pgRNA, HBsAg, and HBcAg proteins. The group was divided according to the control group (transfected with HBV whole genome plasmid) and the Solamargine group (transfected and added 5 μM Solamargine). Northern blot results were consistent with expectations, which again verified that the relative expression level of HBV pgRNA in the Solamargine group was lower than that in the control group (Figure 3A), confirming that Solamargine could significantly inhibit the expression level of HBV pgRNA. Western blot showed that the relative expression of HBcAg protein in the Solamargine group was lower than that in the control group, while there was no significant change in the relative expression of HBsAg protein (Figure 3B). The results indicated that Solamargine decreased the relative expression of HBcAg protein, but had no significant effect on the relative expression of HBsAg protein.

### Solamargine reduces the fluorescence relative expression of HBV core promoter and its series of mutants

**Figure 4:**
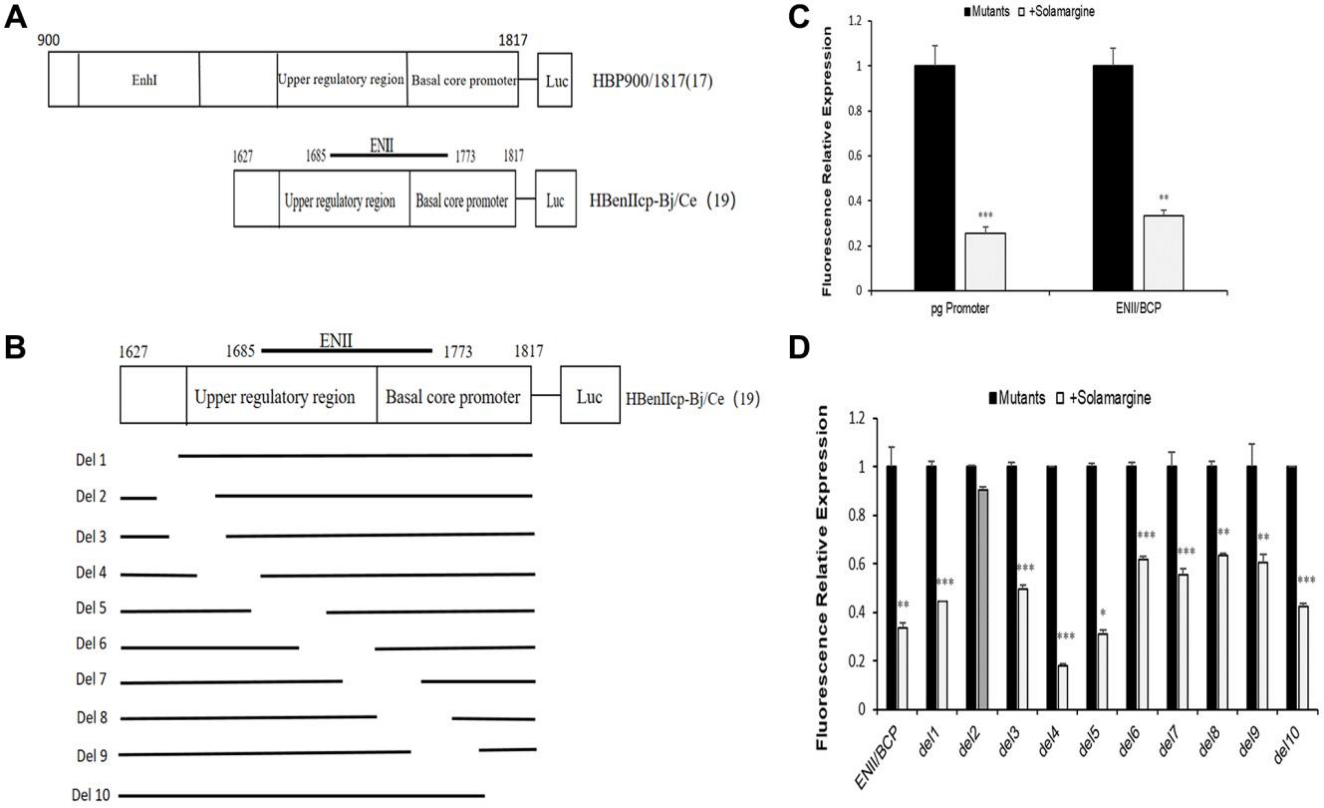
**Effects of Solamargine on fluorescence relative expression of HBV core promoter and its series of mutants.** (**A**) Construction of HBV pgpromoter and ENII/BCP fluorescence reporter vector plasmid by PCR; (**B**) Construct plasmid of ENII/BCP and its series of gene deletion mutants pGL4.74-HBenIIcp-Ce-del1 to pGL4.74-HBenIIcp-Ce-del10 by PCR; (**C**, **D**) Each mutant plasmid was transfected into Huh7 cells, and after 2 d Solamargine (5 μM) was added, and the samples were collected after 1 d culture. HBV pgpromoter and ENII/BCP fluorescence relative expression were detected by sea kidney luciferase assay (**C**). The fluorescence relative expression levels of ENII/BCP and its gene deletion mutants were also detected (**D**). The relative expression level of fluorescence in control group was 1. The results are means±SD of 3 independent experiments. Statistical differences compared with the control are shown. ^*^p < 0.05, ^**^p < 0.01, ^***^p < 0.001, Student’s *t*-test.

The above experimental results all indicate that Solamargine affects the levels of HBV pgRNA and HBcAg protein, which suggests that Solamargine may inhibit viral expression by acting on the core promoter of HBV genome. To test the guess, we constructed HBV pgpromoter fluorescence reporter vector by PCR (pGL4.74-HBpg-Ce, nt900-1817) and on this basis, the ENII/BCP fluorescence reporter vector was also constructed (pGL4.74-HBenIIcp-Ce, nt1627-1817) (Figure 4A).

The groups were divided according to the control group (transfection of HBV pgRNA promoter plasmid), the experimental group (transfection and added Solamargine 5 μM), the control group (transfection with ENII/BCP plasmid) and the experimental group (transfection and added Solamargine 5 μM). The results of the sea kidney luciferase experiment showed that the relative expression of fluorescence in the Solamargine group was lower than that in the control group (*p* < 0.05) (Figure 4C). This told us that Solamargine not only decreased the fluorescence expression level of HBV pgRNA promoter but also the fluorescence expression level of ENII/BCP. Based on the above results, we considered that Solamargine acted on a fragment of HBV core promoter and affected HBV gene expression. Therefore, a series of gene deletion mutants pGL4.74-HBenIIcp-Ce-del1 to pGL4.74-HBenIIcp-Ce-del10 based on the core promoter PGL4.74-HBenIIcp-Ce-del10 were constructed again (Figure 4B). The effect of Solamargine on the relative fluorescence expression of each mutant was detected. The results showed that the relative expression of fluorescence in pGL4.74-HBenIIcp-Ce-del2 group after adding Solamargine had no significant change compared with the control group (*p* > 0.05), while the other mutant groups after adding Solamargine were all lower than the control group (*p* < 0.05) (Figure 4D). This indicated that Solamargine targeted pGL4.74-HBenIIcp-Ce-del2. In general, through the above experiments, we can conclude that Solamargine acts on the HBV core promoter and targeted pGL4.74-HBenIIcp-Ce-del2 fragment (the segment sequence of 5′-GCCCAAGGTCTTACATAAGAGGACTCTTGGACTCT-3′) to suppress HBV replication and expression.

### Solamargine targeted pGL4.74-HBenIIcp-Ce-del2-associated transcription factors to regulate HBV replication

**Figure 5:**
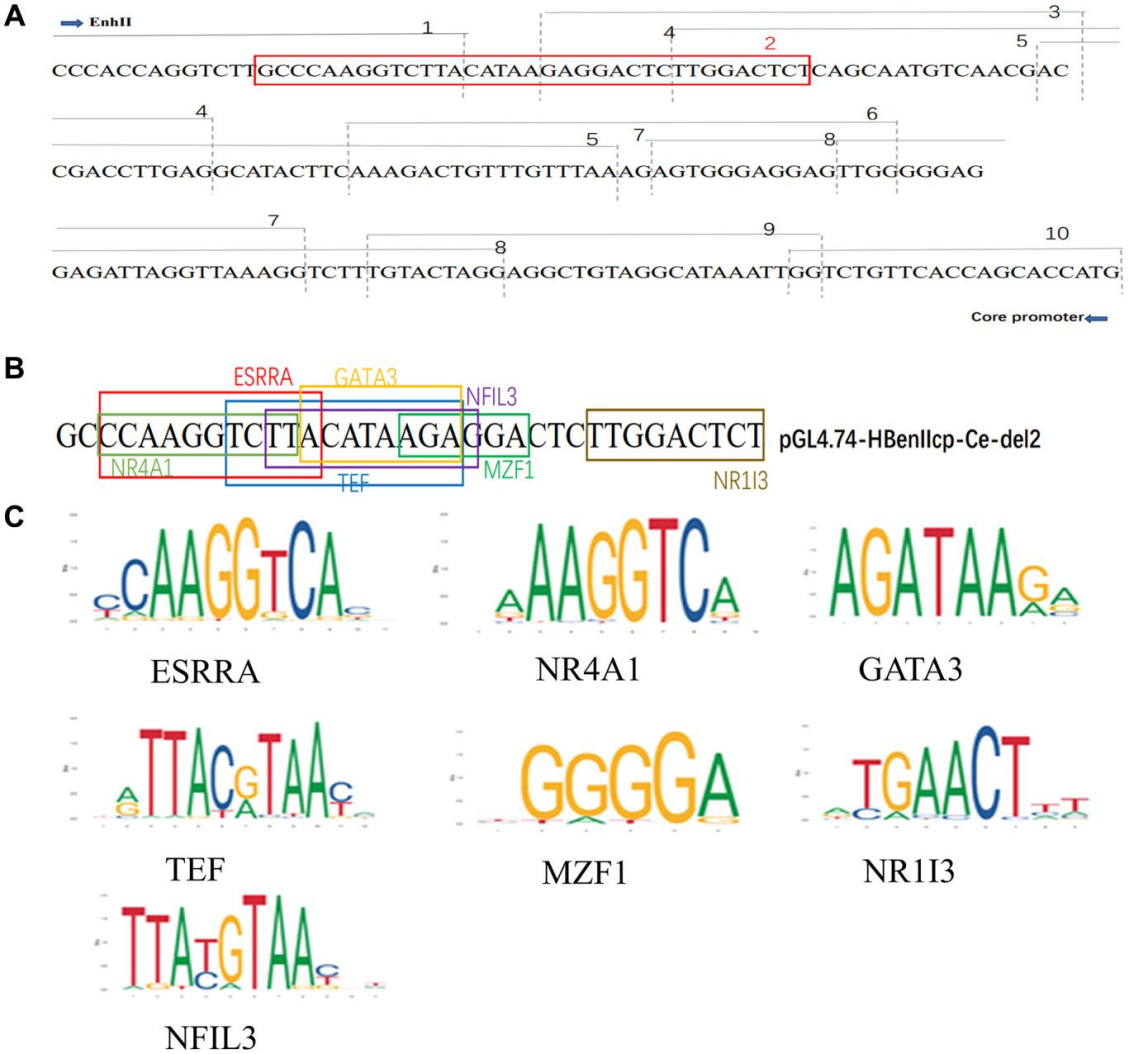
**Solamargine regulates HBV replication by targeting PGL4.74-HBenIIcp-Ce-Del2 related transcription factors.** (**A**) ENII/BCP and a series of deletion mutants from pGL4.74-HBenIIcp-Ce-del1 to pGL4.74-HBenIIcp-Ce-del10 gene sequence. (**B**) The deletion of HBenIIcp-Ce-del1 to pGL4.74-HBenIIcp-Ce-del10 gene sequence. (**C**) The deletion of pGL4.74-HBenIIcp-Ce-del2 DNA sequence and the presence of interacting transcription-related proteins in the sequence.

Multiple liver-enriched and ubiquitous transcription factors regulate viral transcription and replication by targeting promoter and enhancer regions [13]. These results demonstrated that Solamargine acted on the deletion fragment of HBV core promoter pGL4.74-HBenIIcp-Ce-del2, and we speculated that the DNA sequence in this region might interact with a transcription factor to regulate HBV replication (Figure 5A). Thus, identify transcription factor binding sites from the JASPAR database (https://jaspar.genereg.net). The transcription factors binding to the specific sites of the DNA sequence of this segment were analyzed. Among them, seven transcription-related proteins ESRRA, NR4A1, TEF, MZF1, GATA3, NR1I3, and NFIL3 were predicted to be the specific binding factors of the DNA sequence of this target gene (Figure 5B, 5C).

### Solamargine inhibited HBV transcription by interacting with the transcription factor MZF1

**Figure 6:**
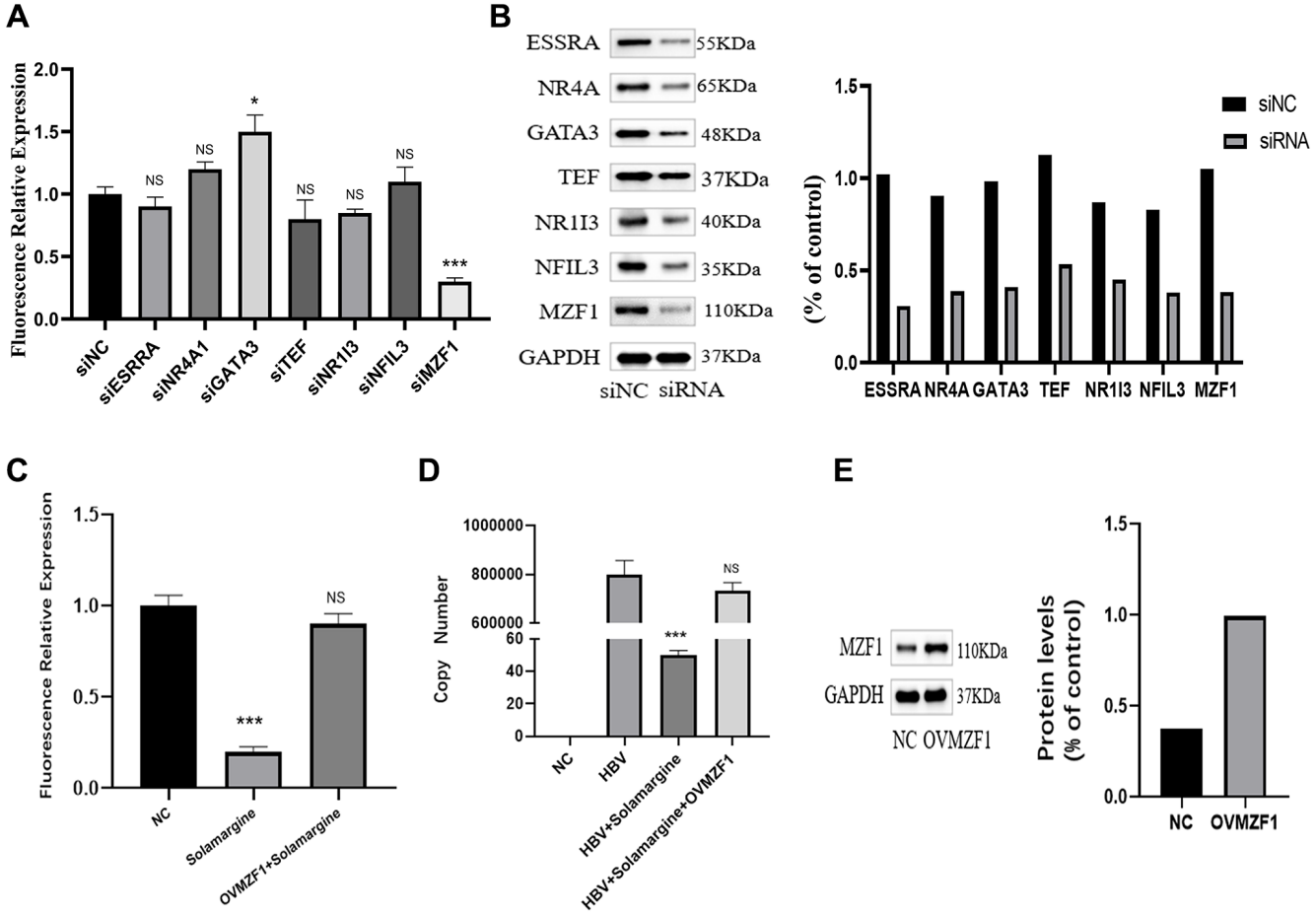
**Solamargine interacts with transcription factor MZF1 to inhibit the activity of pgpromoter and the replication of hepatitis B virus.** (**A**) HBV pgpromoter luciferase assay vector was transfected into Huh7 cells. 2 d later, siRNA of potential banding sites was also transfected, the luciferase relative expression in every group was detected. (**B**) Protein levels of banding sites in Huh7 cells. GAPDH was used to normalize protein levels. (**C**) HBV pgpromoter luciferase assay vector was transfected into Huh7 cells for 2d, then one group was added Solamargine 5 μM, the other group overexpressed MZF1 simultaneously and the luciferase relative expression was detected. (**D**) HBV plasmid was transfected into Huh7 cells, 2 d later Solamargine was added into the cells, the other group overexpressed MZF1 simultaneously. Samples were collected after 1 d culture, and the HBV DNA copy number of supernatant was detected by PCR. (**E**) MZF1 level in Huh7 cells. GAPDH was used to normalize protein levels. All assays were performed in triplicate and results are presented as means ± SD. ^*^p < 0.05, ^**^P < 0.01, ^***^P < 0.001, Student’s *t*-test.

To further confirm which transcription factor Solamargine binds to, we knocked down every potential binding site in Huh7 cells (Figure 6B), and then HBV pgRNA promoter fluorescence reporter vector was transfected into the cells. Finally, the relative fluorescence expression was respectively detected by sea kidney luciferase assay (Figure 6A). The results showed that only knocking down MZF1 could inhibit pgRNA promoter activity. Therefore, we boldly hypothesized that Solamargine interacted with MZF1 to inhibit the activity of pgRNA promoter to act as an antiviral. Next, to test this hypothesis, we transfected pgRNA promoter fluorescence reporter vector plasmid into Huh7 cells, overexpressed the intracellular transcription factor MZF1, added Solamargine, and the fluorescence relative expression was detected (Figure 6C). The result showed that the overexpression of MZF1 inhibited the effect of Solamargine on pgRNA promoter. Lastly, we transfected HBV plasmid into Huh7 cells, overexpressed MZF1, and added Solamargine, the relative expression level of HBV DNA in the supernatant was detected (Figure 6D, 6E). Similarly, the overexpression of MZF1 also inhibited the inhibitory effect of Solamargine on hepatitis B virus. In conclusion, these results told us that Solamargine could interact with transcription factor MZF1 to inhibit the activity of pgRNA promoter and the replication of hepatitis B virus.

## DISCUSSION

Chronic hepatitis B virus infection is still a global public health problem, and the elimination and cure of chronic hepatitis B virus are facing great challenges. At present, “HBV cure” includes two types: (1) Complete cure, serum HBsAg negative, HBV cccDNA in hepatocytes, and serum HBV DNA completely cleared; (2) Functional cure, continuous clearance of HBsAg or serological conversion, continuous undetectable HBV DNA, and reduced risk of HCC [14]. The continuous and stable existence of cccDNA in hepatocytes is the main cause of long-term persistent and chronic infection. The accumulation of cccDNA promotes the persistence of HBV replication and is the source of viral rebound after antiviral therapy [15, 16]. However, for most patients, current antiviral drugs only reduce liver tissue inflammation and fibrosis and delay the progression of complications by inhibiting viral replication, and cannot clear HBV cccDNA. The complete cure of HBV is difficult to achieve, and indefinite treatment is needed to maintain viral inhibition level [17]. Therefore, the current ideal therapeutic goal of chronic hepatitis B is to achieve a “functional cure” of viral infection to stop the natural course of HBV and prevent complications of chronic liver disease [18, 19]. Nowadays, acting on the life cycle of viruses to inhibit virus replication has always been the research direction of antiviral drugs. New treatment strategies for chronic hepatitis B currently under development fall into two main categories: (1) Direct antivirals that disturb the life cycle of the virus: Entry inhibitors targeting NTCP, inhibitors or regulators that inhibit pre-genomic RNA assembly into nucleated capsids, silencing cccDNA, gene editing techniques, HBV Pol inhibitors, RNA interference (RNAi), HBsAg secretion inhibitors and so on [20]; (2) Immunotherapy methods to modulate host immune response: Toll-like receptors mediating immune response activation, therapeutic vaccines, monoclonal antibodies, and targeted checkpoint inhibitors [21].

Solanum nigrum L is an annual herb of the genus Solanaceae. The whole plant contains a variety of alkaloids, polyphenols, saponins, lignans, polysaccharides, etc. In addition, it also contains vitamins, nutritional minerals, amino acids, and other nutrients. It has anti-inflammatory, anti-tumor, immune regulation, antipyretic analgesia, antihypertensive and other effects [22, 23]. Previous studies have shown that solenodon is a suitable source of steroid alkaloids used in the synthesis of some steroid hormones. Solamargine is the main steroid glucoside alkaloid present in immature fruits [24]. The alkaloids in Solamargine are the main active components of anti-tumor, and Solamargine, as the main components of alkaloids, has been proven to have obvious inhibitory effects on a variety of malignant tumors [24], this study also demonstrated that Solamargine inhibited the proliferation of HCC and effectively induced apoptosis and autophagy of HCC cells *in vitro* and *in vivo*, clarifying the mechanism of action and providing a research direction for effective candidate drugs for HCC treatment in the future. Solamargine inhibited the activity of five gastric cancer cell lines in a dose-dependent manner, induced significant changes in cell morphology, and inhibited the progression of gastric cancer by regulating the expression of lncNEAT1_2 through MAPK pathway [25]. Solamargine not only inhibits the progression of liver cancer and stomach cancer but also has obvious anticancer activity against cervical cancer, lung cancer, breast cancer, colorectal cancer, nasopharyngeal cancer, prostate cancer, and so on, with significant curative effects and few side effects [26–29]. However, there are few studies on the effect of Solamargine on HBV, only simple isolation from Solamargine and identification of anti-HBV activity, further studies on specific targets and mechanisms of action have not been reported.

In this study, we successfully established a stable HBV genome expression cell line based on the pEB-puromycin vector (right picture of Figure 1A), which could stably replicate the HBV genome of genotype Ce. In HBV-infected hepatocytes, the viral genome cccDNA, which serves as a transcription template, exists as a stable free body and cannot be integrated [30]. In our new pEB-puromycin stable cell line, HBV DNA transcription is via HBV’s promoter independent of chromosomes, whereas in HepG2.2.15 cell line, HBV DNA transcription is not dependent on chromosomes, HBV genes are embedded in chromosomes and transcribed by chromosomal promoters. In general, in the pEB-puromycin gene model, HBV did not integrate into the cell chromosome after infection but dissociated outside the cell chromosome, which is closer to the real model of human infection with the hepatitis B virus. This research group has published relevant articles before [31, 32]. First, we used stable cell lines to screen out compounds with high anti-HBV activity. There are ten compounds with different levels of antiviral activity. The results showed that Solamargine significantly reduced the expression level of HBV pgRNA, suggesting that Solamargine inhibited HBV replication and transcription. Next, we further verified the effect of this compound on HBV. Studies have shown that Solamargine can effectively inhibit the expression of HBV DNA in the supernatant of HBV-infected cells. Northern blot confirmed that Solamargine significantly inhibited HBV pgRNA expression. Western blot results indicated that the compound inhibited HBcAg protein expression, but had no significant effect on the relative expression of HBsAg protein.

The core promoter plays an important role in viral replication, directing the initiation of transcription as well as the synthesis of pc/pgRNA, which not only acts as a template for viral reverse transcription but also can be translated into HBcAg and polymerase [33]. Interestingly, our results have demonstrated that Solamargine inhibits HBV pgRNA expression and HBcAg protein expression, both of which are controlled by the core promoter of the HBV genome. So, we hypothesized that Solamargine inhibited viral replication and expression by targeting HBV core promoter. For this purpose, we constructed HBV pgRNA promoter and ENII/BCP fluorescence reporter vectors. The results showed that Solamargine not only decreased the fluorescence expression level of the pgRNA promoter but also the core promoter. It is suggested that the compound targets HBV core promoter. To further explore its specific targets, we constructed a series of gene deletion mutants pGL4.74-HBenIIcp-Ce-del1 to pGL4.74-HBenIIcp-Ce-del10 based on the ENII/BCP and then detected the fluorescence expression. The results showed that in pGL4.74-HBenIIcp-Ce-del1 to pGL4.74-HBenIIcp-Ce-del10 groups with gene deletion, the relative fluorescence expression of del2 group had no significant change after adding Solamargine, but was lower than that of other mutant groups. These results indicated that Solamargine targeted pGL4.74-HBenIIcp-Ce-del2 fragment to regulate HBV replication.

Transcription factors include DNA-binding domains and effector domains or transcriptional regulatory domains that provide nuclear localization signals [34]. Previous studies have shown that liver-rich transcription factors, especially the liver nuclear factor family, play an important role in the regulation of HBV replication and transcription, and can enhance the activity of preS, CP, and ENII. Of course, several other ubiquitous transcription factors are also crucial [35]. We have demonstrated that Solamargine targets the missing fragment of HBV core promoter pGL4.74-HBenIIcp-Ce-del2. To further elucidate its mechanism of action, we analyzed transcription factors from the JASPAR database (https://jaspar.genereg.net), which interacted with the segment of DNA sequence. Among them, seven transcription-related proteins ESRRA, NR4A1, TEF, MZF1, GATA3, NR1I3, and NFIL3 were predicted to be specific binding factors of the DNA sequence of this target gene. To verify which transcription factor Solamargine binds to, we transfected siRNA of potential binding sites and pgRNA promoter fluorescence reporter vector into Huh7 cells successively and detected fluorescence expression. The result showed that only knocking down MZF1 could inhibit the activity of the pgRNA promoter. To further confirm whether Solamargine interacts with MZF1 to inhibit HBV, we overexpressed MZF1 in Huh7 cells. And the results showed that the overexpression of MZF1 could inhibit the antiviral effect of Solamargine.

## MATERIALS AND METHODS

Materials and reagents Huh7 cells (All the cells used in the experiment were detected by mycoplasma and identified by STR) and HBV Ce genotype expression plasmid (pUC-HB-CepUC19-HBV-Ce, which contains the 1.24-fold HBV genome) were donated by Dr. Mizokami (National Center for Global Health and Medicine, Japan); DMEM High sugar base medium (11965092, DMEM, Gibco, MA, USA), 1X PBS Buffer Solution (10010023, PBS Gibco, MA, USA) and Fetal calf serum (16140071, FBS, Gibco, MA, USA) all purchased from hermo Fisher Scientific (MA, USA); natural compound library was bought from MCE Company NJ, USA), concentration: Natural compound 1ul: DMEM medium 2 ml, the final concentration of 5 μM, according to this ratio of configuration; total RNA extraction kits(RE-03113) for cultured cells were purchased from Foregene (Chengdu, China); reverse transcription kit(R323-01) was purchased from Vazyme (Nanjing, China), and fluorescence quantification kit (PM101-01) was purchased from MCE (Shanghai, China; Lipo3000 (L3000075) and Lipo2000 transfection reagents (11668030) were purchased from Thermo Fisher Scientific (MA, USA); the Radio Immunoprecipitation Assay lysate (RIPA, R0020) as well as PMSF(P0100) were purchased from Solarbio Corporation (Beijing, China), Protein marker (26616) was purchased from Thermo Fisher Scientific (MA, USA); BCA kit (P0010S), SDS-PAGE gel configuration kit (P0012A), ECL chemiluminescence kit (PO018S) were purchased from Guangzhou Baiyun Sky Company, China; sea kidney luciferase reporter gene detection kit (E6881) was bought from Promega (WI, USA). GAPDH antibody was purchased from ABclonal Biological Company, Wuhan, China (A19056). GAPDH was bought from Santa Cruz Biotechnology, TX, USA (sc-77724). HRP-conjugated secondary antibody (7076P2, Cell Signaling Technology (MA, USA). Goat anti-human HBsAg antibody was purchased from Biorbyt Biotechnology Company (UK) (orb10774). HBcAg antibody was bought from Abcam (UK) (EPR28251-34). ESRRA antibody was bought from the Family Biology official website (DF7775). MZF1 antibody was bought from Abcam, UK (ab64866). NR4A1 antibody was bought from Wuhan Huamei Biological Engineering Company, China (CSB-PA179677). GATA3 antibody was bought from Hangzhou BAILing Biotechnology, China (BX50252). NR1I3 antibody was bought from Solarbibo Technology, , China (Z35320). TEF4 antibody was purchased from Shanghai Caiyou Technology Company, China (s-8212R). NFIL3 antibody was bought from Abcam compan, UK (ab230090)

### Methods

#### Cell culture, transfection

Huh7 cells were cultured in a culture dish containing 10% fetal bovine serum and 100 mg/L penicillin/ streptomycin in DMEM, and placed in a 5% CO_2_ incubator at 37°C to observe cell growth. After having filled 10cm dishes, cells were seeded in 24-well plates, and after the cell growth density reached 80–90%, the plasmid was transfected into Huh7 cells with Lipo3000 and P3000 transfection reagent.

#### RNA extraction, RT-qPCR

After separate transfection of cells as described above, cells were harvested, RNA was extracted with cultured cell total RNA extraction kit, RNA was reverse transcribed to complementary DNA (cDNA) with reverse transcription kit, and fluorescence quantification system configuration with SYBR Green qPCR Master Mix: 2 × SYBR green master mix 10 μl, ROX 0.4 μl, primer 0.4 μl, cDNA 2 μl, DEPC-H2O 6.8 μl, reaction conditions: 95°C 30 S; 95°C 5 S; 50°C 40 S, 40 cycles; 95°C 15 S, 60°C 30 S, 95°C 15S. β-actin as the internal reference and amplified in a quantitative PCR instrument to detect pgRNA content. Then the 2^−ΔΔCt^ method was used to calculate the results. The lengths and sequences of the primers are shown in Table 2.

#### Northern blot, western blot

Total RNA was extracted, treated with DNase I and RNase inhibitors, and separated in 1×3-(N-morpholino) propane sulfonate (MOPS) buffer (20 mM MOPS, 5 mM sodium acetate, and 2 mM EDTA) at 60 V for 3 h; the sample was transferred to a nylon membrane with a 20× SSC transfer buffer and crosslinked to the membrane by ultraviolet light (120 mJ/cm^2^); At 68°C, the DIG Northern Starter Kit reagent was hybridized overnight with DIG-11-UTP DIG-labeled RNA probes; After the CDP-Star detection reagent was added, the DIG-labeled probe on the imprinted membrane was exposed and imaged. After sample collection, Cell samples in 24-well plates were collected and each well was added 75 ul RIPA of lysate + PMSF (100:1) was added to each well, and 20 μl of lysate was taken to determine the protein concentration according to the BCA assay, the 20 μl of boiled protein samples were subjected to PAGE gel electrophoresis, and the electrophoretic proteins were transferred to a Polyvinylidene fluoride (PVDF) membrane, the PVDF membrane was immersed in TBST containing 5% skim milk powder in a 37°C shaker 55 turn and blocked for 1 h, The membrane was cut according to the protein molecular weight and marker instructions, the corresponding PVDF membrane bands were immersed in the primary antibody solution, the primary antibody ratio was 1:1000 (3 ml 5% skim milk powder + 3 ul primary antibody), incubated at 4°C overnight, and then the film was washed in TBST; horseradish peroxidase-labeled secondary antibodies were added, The proportion of secondary antibody was 1:5000 (5 ml 5% skim milk powder + 1 ul secondary antibody) was incubated at room temperature for 90 min, TBST film, the PVDF film soaked in the luminous color solution to the band color clear, the film in the chemiluminescence scanner scan, photo, preservation.

#### Construct plasmid mutant

The HBV genotype pUC-HB-Ce contains 1.24 times HBV genome, and its sequence is derived from the common sequence of the HBV genotype Ce subtype. The sequence of the genome is shown in Supplementary Material 1. To construct PGL4.74-HBpg-Ce according to the most common nucleotides in HBV genotype Ce, the synthetic fragment corresponding to nt 900-1817 region of HBV genome was inserted into the upstream of the pGL4.74 luciferase reporter gene after digestion by KpnI and HindIII. To construct PGL4.74-HBenIICP-Ce, the synthetic fragment corresponding to nt 1627-1817 region was amplified by PCR based on HBV pgRNA promoter fluorescence reporter vector, and cloned to pGL4.74 by the above method. Besides, a series of deletion mutants pGL4.74-HBenIIcp-Ce-del1 to pGL4.74-HBenIIcp-Ce-del10 were constructed according to the above method. The del 1 (deletion of nt.1627-1657), The del 2 (deletion of nt.1642-1677), The del 3 (deletion of nt.1659-1689), The del 4 (deletion of nt.1674-1704), The del 5 (deletion of nt.1689-1726), The del 6 (deletion of nt.1710-1742), The del 7 (deletion of nt.1726-1736), The del 8 (deletion of nt.1721-1753), The del 9 (deletion of nt.1738-1770), del 10 (deletion of nt.1785-1817) were constructed.

#### Luciferase reporter assay

Cells were transfected with HBV pgRNA promoter and ENII/BCP-driven sea kidney luciferase reporter plasmid. After 48h transfection, cells were collected, culture medium was removed, and 1 × PBS solution was washed twice. Add diluted lysate 120 μL and shake at room temperature for 15 min; The supernatant was collected into the EP tube at 4°C, with a radius of 10 cm, and centrifugation at 10000 r/min for 3 ~ 5 min. Open the enzyme marker in advance, select the luminance, and set the parameters. Prepare the working liquid (the product ratio of liquid C to liquid B is 1:49). The 50 μL cell lysate was added into the 96-well plate and 100 μL working fluid. The luminescence value of luciferase was determined in the enzyme-labeled instrument. The luminescence value per μg protein represented the HBV pgRNApromoter and ENII/BCP luciferase activities.

### Statistical analysis

The above experiments were repeated more than three times, and SPSS 22.0 statistical software was used for analysis. The experimental data in this study were measurement data with normal distribution. Independent sample *t*-test was used for comparison between two groups, one-way analysis of variance was used for comparison between multiple groups, and LSD test was used for pound-to-group comparison. *p* < 0.05 was considered statistically significant.

### Data availability

The data of this study are available from the corresponding author upon request.

## Supplementary Materials

Supplementary Material 1

Supplementary Figure 1
