# Supplementary material for: Solamargine acts as an antiviral by interacting to MZF1 and targeting the core promoter of the hepatitis B virus gene
Source: Aging (Albany NY). 2024 Aug 10;16(15):11668–82. doi: 10.18632/aging.206047 (PMC11346786; doi:10.18632/aging.206047)
Supplement: Supplementary Material 1 [file aging-16-206047-s001.pdf]

SUPPLEMENTARY MATERIAL

Supplementary Material 1. The sequence of pUC-HB-CepUC19-HBV-Ce.

The sequence of the genome which contained 1.24-fold HBV genome AATTTGTCCTGGCTATCGCTGGATGTGT
CTGCGGCGTTTTATCATATTCCTCTTCATCCTGCTGCTATGCCTCATCTTCTTGTTGGTTCTTCTGGACTACC
AAGGTATGTTGCCCGTTTGTCCTCTACTTCCAGGAACATCAACTACCAGCACGGGACCATGCAAGACCTGC
ACGATTCCTGCTCAAGGAACCTCTATGTTTCCCTCTTGTTGCTGTACAAAACCTTCGGACGGAAACTGCACT
TGTATTCCCATCCCATCATCCTGGGCTTTCGCAAGATTCCTATGGGAGTGGGCCTCAGTCCGTTTCTCCTGG
CTCAGTTTACTAGTGCCATTTGTTCAGTGGTTCGTAGGGCTTTCCCCCACTGTTTGGCTTTCAGTTATATGGA
TGATGTGGTATTGGGGGCCAAGTCTGTACAACATCTTGAGTCCCTTTTTACCTCTATTACCAATTTTCTTTTG
TCTTTGGGTATACATTTGAACCCTAATAAAACCAAACGTTGGGGCTACTCCCTTAACTTCATGGGATATGTA
ATTGGAAGTTGGGGTACTTTACCGCAGGAACATATTGTACTAAAACTCAAGCAATGTTTTCGAAAACTGCC
TGTAAATAGACCTATTGATTGGAAAGTATGTCAAAGAATTGTGGGTCTTTTGGGCTTTGCTGCCCCTTTTAC
ACAATGTGGCTATCCTGCCTTAATGCCTTTATATGCATGTATACAATCTAAGCAGGCTTTCACTTTCTCGCC
AACTTACAAGGCCTTTCTGTGTCAACAATATCTGAACCTTTACCCCGTTGCCCGGCAACGGTCAGGTCTCTG
CCAAGTGTTTGCTGACGCAACCCCCACTGGATGGGGCTTGGCCATAGGCCATCGGCGCATGCGTGGAACCT
TTGTGGCTCCTCTGCCGATCCATACTGCGGAACTCCTAGCAGCTTGTTTTGCTCGCAGCCGGTCTGGAGCGA
AACTTATCGGGACTGACAACTCTGTTGTCCTCTCTCGGAAATACACCTCCTTTCCATGGCTGCTAGGGTGTG
CTGCCAACTGGATCCTGCGCGGGACGTCCTTTGTCTACGTCCCGTCGGCGCTGAATCCCGCGGACGACCCG
TCTCGGGGCCGTTTGGGCCTCTACCGTCCCCTTCTTCATCTGCCGTTCCGGCCGACCACGGGGCGCACCTCT
CTTTACGCGGTCTCCCCGTCTGTGCCTTCTCATCTGCCGGACCGTGTGCACTTCGCTTCACCTCTGCACGTCG
CATGGAGACCACCGTGAACGCCCACCAGGTCTTGCCCAAGGTCTTACATAAGAGGACTCTTGGACTCTCAG
CAATGTCAACGACCGACCTTGAGGCATACTTCAAAGACTGTTTGTTTAAGGACTGGGAGGAGTTGGGGGA
GGAGATTAGGTTAAAGGTCTTTGTACTAGGAGGCTGTAGGCATAAATTGGTCTGTTCACCAGCACCATGCA
ACTTTTTCACCTCTGCCTAATCATCTCATGTTCATGTCCTACTGTTCAAGCCTCCAAGCTGTGCCTTGGGTGG
CTTTGGGGCATGGACATTGACCCGTATAAAGAATTTGGAGCTTCTGTGGAGTTACTCTCTTTTTTGCCTTCT
GACTTCTTTCCTTCTATTCGAGATCTCCTCGACACCGCCTCTGCTCTGTATCGGGAGGCCTTAGAGTCTCCG
GAACATTGTTCACCTCACCATACAGCACTCAGGCAAGCTATTCTGTGTTGGGGTGAGTTGATGAATCTGGC
CACCTGGGTGGGAAGTAATTTGGAAGACCCAGCATCCAGGGAATTAGTAGTCAGCTATGTCAATGTTAATA
TGGGCCTAAAAATCAGACAACTATTGTGGTTTCACATTTCCTGTCTTACTTTTGGAAGAGAAACTGTTCTTG
AGTATTTGGTGTCTTTTGGAGTGTGGATTCGCACTCCTCCCGCTTACAGACCACCAAATGCCCCTATCTTAT
CAACACTTCCGGAAACTACTGTTGTTAGACGACGAGGCAGGTCCCCTAGAAGAAGAACTCCCTCGCCTCGC
AGACGAAGGT.
