# Supplementary material for: Solamargine acts as an antiviral by interacting to MZF1 and targeting the core promoter of the hepatitis B virus gene
Source: Aging (Albany NY). 2024 Aug 10;16(15):11668–82. doi: 10.18632/aging.206047 (PMC11346786; doi:10.18632/aging.206047)
Supplement: Supplementary Figure 1 [file aging-16-206047-s002.pdf]

SUPPLEMENTARY FIGURE

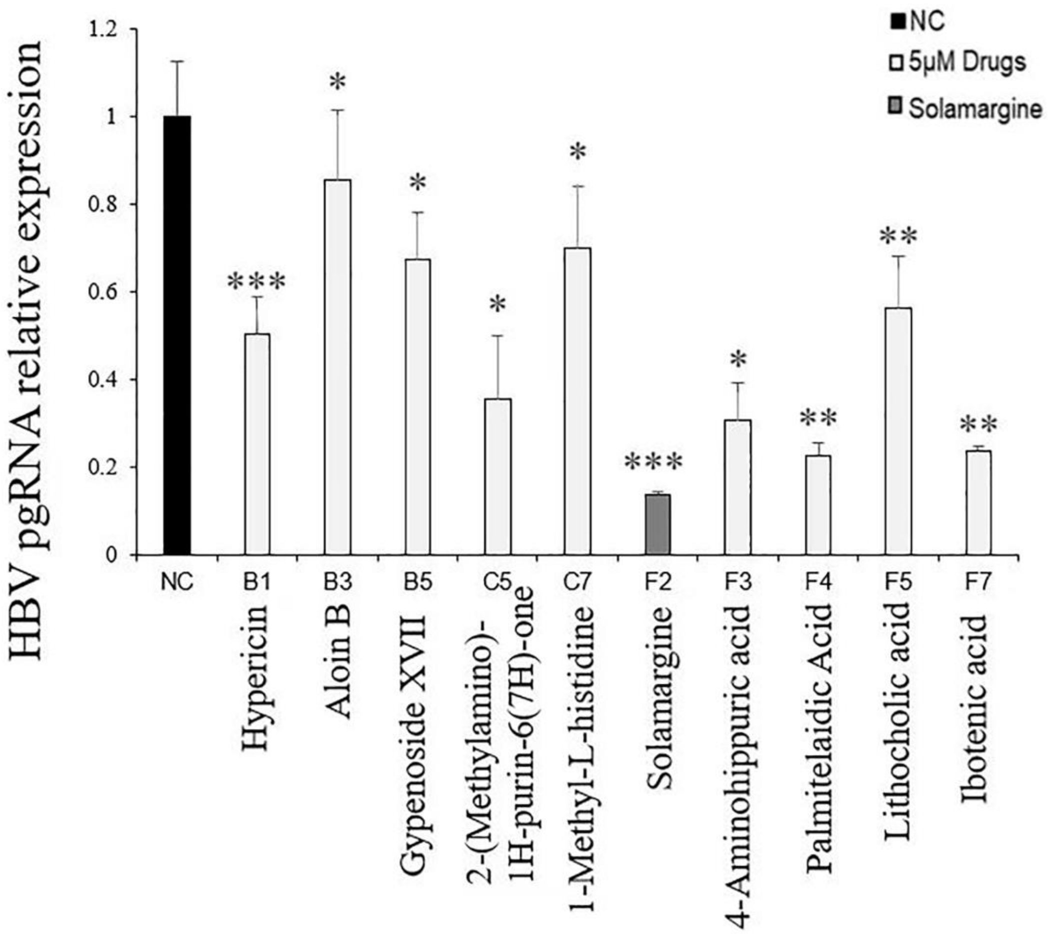

Supplementary Figure 1. Natural compounds exhibited anti-HBV pgRNA effect.
